# PET Testing Has Utility in the Prescription of Peritoneal Dialysis: Commentary

**DOI:** 10.34067/KID.0000000000000409

**Published:** 2024-04-04

**Authors:** Ramesh Saxena

**Affiliations:** Division of Nephrology, Department of Internal Medicine, UT Southwestern Medical Center, Dallas, Texas

**Keywords:** dialysis, peritoneal dialysis, peritoneal membrane

Structural and functional integrity of the peritoneal membrane (PM) is paramount for the efficacy of peritoneal dialysis (PD). Hence, it makes sense to assess characteristics of PM in individuals on PD. The standardized peritoneal equilibration test (PET) using 4-hour exchange with 2 L of 2.5% dextrose dialysate is the common clinical tool used to evaluate PM. Dialysate-to-plasma ratios of creatinine at 2 and 4 hours and the ratios between glucose concentrations in the dialysate at 2 and 4 hours and at the beginning of the test (D2/D0 and D4/D0, respectively) are used to characterize the peritoneum into slow, average, or fast peritoneal solute transfer rate (PSTR) categories. Alternatively, a modified PET using 4.25% dextrose dialysate can be performed. The 4.25% PET allows the assessment of sodium sieving, an effect of free water transfer across transendothelial ultra-small pores (Aquaporin-1). The current International Society of Peritoneal Dialysis guidelines recommend conducting a PET, preferably using 4.25% dextrose, early after PD initiation and to repeat it when clinically indicated.^1^

In this issue of *Kidney360*, Drs. Schreiber and Yang and Bargman convincingly discuss contrasting perspectives on the utility of PET testing in prescription of PD.^2,3^ Schreiber highlights the importance of PET in identifying patients at risk of hypervolemia and PD loss that should lead to closer follow-up, development of mitigating strategies, and prescription modification.^2^ Moreover, he recommends exploration of innovative techniques based on artificial intelligence and machine learning to devise a virtual PET, particularly for centers that have logistic challenges in performing PET. Contrarily, Yang and Bargman argue that PET is not necessary for PD prescription, citing its limitations, including cost (both economic and staffing), patient inconvenience, lack of convincing outcome data, and potential errors during conductance of the test.^3^ Moreover, they argue that PD can be appropriately prescribed on the basis of clinical assessment alone without using PET data.

The PD care should be goal directed with emphasis on realization of life goals of the person and effective management of volume status, comorbidities, and nutritional status.^4^ In the context of the holistic approach of providing comprehensive care, the role of PD *per se* is to remove uremic solutes, provide ultrafiltration (UF) to attain euvolemia, and concurrently curtail metabolic cost of PD by minimizing glucose load.

Whereas small solute removal, measured as KT/Vurea, reflects only one of the numerous aspects of dialysis; it remains a major quality metric for dialysis facilities in the United States, being austerely monitored by regulatory agencies. Several computer-based models using PET data have emerged to generate PD prescription that will meet the stipulated KT/Vurea target. However, growing de-emphasis on small solute clearance target makes the role of PET less relevant in this aspect. Moreover, PET data are not available at the time of PD start, and initial PD prescriptions are empirically generated, on the basis of the size of the individual and presence or absence of residual kidney function. Subsequent changes in PD prescription can be made on the basis of clinical assessment without assistance from PET data.

Hypervolemia is widespread among patients on PD and is strongly associated with high risk of mortality, cardiovascular events, and PD technique failure.^1,4^ Achievement of euvolemia, hence, is a major goal of high-quality PD prescription. Dialysates with high glucose concentrations are commonly used to optimize UF. Consequently, metabolic effects of glucose absorption have become a growing concern, particularly with the rising number of patients with diabetes starting PD. PET data can be used to estimate daily glucose absorption from PD dwells. The ensuing caloric load can then be incorporated in the daily dietary caloric intake of the individual to optimize diabetes management.^5^

Among individuals initiating PD, there is a large variation in PSTR, which can be a determinant of long-term PD outcomes. Indeed, a linear association between dialysate-to-plasma creatinine ratio and mortality and hospitalization was observed in a contemporary cohort of patients treated with automated PD (APD).^6^ Moreover, PSTR can change over time because of molecular and structural changes in the peritoneum from prolonged exposure to contemporary dialysis solutions. Typically, there is upsurge in small solute transport that causes rapid glucose absorption and ensuing reduction in crystalloid osmotic gradient and aquaporin-mediated free water transfer that can incite UF insufficiency and high glucose load.^7^ While UF volume of <400 ml at 4 hours with 4.25% PET is the recommended diagnostic test to recognize patients at risk to develop UF insufficiency, volume measurement can be inaccurate for a variety of reasons.^1^ Sodium sieving at 1 hour with 4.25% PET may be a more accurate measure to estimate daily UF volume and identify patients at risk to develop UF insufficiency.^8^ Furthermore, progressive loss of sodium sieving may predict development of the encapsulating peritoneal sclerosis, a rare but serious complication of PD.^9^ Switching patients from continuous ambulatory PD (CAPD) to APD and using icodextrin for long daytime dwell can partially mitigate poor outcomes due to hypervolemia observed among individuals with high PSTR.^10,11^ Recently, a conceptual bimodal approach of using short dwells with high-glucose dialysate alternating with long dwells with low or no glucose PD solutions was proposed on the basis of PSTR data from an extended three-pore model to improve UF and solute clearance and reduce metabolic cost among patients on APD.^12^ Another recent study used a Carry Life UF device to maintain a steady glucose dose of 11 g/h among patients on CAPD with high PSTR and demonstrated higher sodium and UF efficiency/g glucose uptake compared with the standard CAPD dwells.^13^

There are several limitations to PET testing. Because the test requires several steps, errors at any stage can confound the PET results. For instance, incomplete drainage of overnight drain can overestimate UF volume. Similarly, the presence of overfill volume in commercially available dialysate bags may cause imprecise UF estimation. Using a standard 2-L fill volume may inaccurately estimate PSTR in very large or smaller individuals. In addition, glucose correction may be needed in patients with hyperglycemia. Improper specimen processing by the dialysis or laboratory staff or mishandling during transit can mar the results. Furthermore, PET testing is time-consuming, costly, and labor-intense. Consequently, many centers with limited resources may not be able to perform a PET study.

In summary, while PET is not necessary to generate or modify a PD prescription, it can provide valuable information to identify patients at risk of UF insufficiency, high glucose load, PM damage, and encapsulating peritoneal sclerosis. Moreover, PET data can be used to estimate daily glucose load. Strategies can be implemented to improve UF while reducing metabolic cost of a PD prescription and improve overall PD outcomes. Hence, whenever possible, PET testing should be performed, notwithstanding its limitations.
